# Respiratory rate variability in sleeping adults without obstructive sleep apnea

**DOI:** 10.14814/phy2.12949

**Published:** 2016-09-05

**Authors:** Guillermo Gutierrez, Jeffrey Williams, Ghadah A. Alrehaili, Anna McLean, Ramin Pirouz, Richard Amdur, Vivek Jain, Jalil Ahari, Amandeep Bawa, Shawn Kimbro

**Affiliations:** ^1^ Pulmonary, Critical Care and Sleep Medicine Division The George Washington University MFA Washington District of Columbia; ^2^ Department of Surgery The George Washington University MFA Washington District of Columbia

**Keywords:** Monitoring, obstructive sleep apnea, respiratory rate variability, spectral analysis

## Abstract

Characterizing respiratory rate variability (RRV) in humans during sleep is challenging, since it requires the analysis of respiratory signals over a period of several hours. These signals are easily distorted by movement and volitional inputs. We applied the method of spectral analysis to the nasal pressure transducer signal in 38 adults with no obstructive sleep apnea, defined by an apnea‐hypopnea index <5, who underwent all‐night polysomnography (PSG). Our aim was to detect and quantitate RRV during the various sleep stages, including wakefulness. The nasal pressure transducer signal was acquired at 100 Hz and consecutive frequency spectra were generated for the length of the PSG with the Fast Fourier Transform. For each spectrum, we computed the amplitude ratio of the first harmonic peak to the zero frequency peak (H_1_/DC), and defined as RRV as (100 − H_1_/DC) %. RRV was greater during wakefulness compared to any sleep stage, including rapid‐eye‐movement. Furthermore, RRV correlated with the depth of sleep, being lowest during N3. Patients spent most their sleep time supine, but we found no correlation between RRV and body position. There was a correlation between respiratory rate and sleep stage, being greater in wakefulness than in any sleep stage. We conclude that RRV varies according to sleep stage. Moreover, spectral analysis of nasal pressure signal appears to provide a valid measure of RRV during sleep. It remains to be seen if the method can differentiate normal from pathological sleep patterns.

## Introduction

Heart rate variability (HRV) (Tobaldini et al. 2013) has been used to describe sleep‐related cardiovascular autonomic alterations. Conversely, the physiological consequences of respiratory rate variability (RRV) during sleep are poorly understood (Tobin et al. 1983; Newton et al. 2014). A major impediment to a greater understanding of RRV has been the lack of a “user‐friendly” clinical technique capable of characterizing periodic alterations in respiratory pattern.

Heart rate variability is readily ascertained from analysis of the ECG, a periodic signal of distinctive morphology. On the other hand, defining RRV in sleep is challenging since it requires the accurate and continuous measurement of airway flow or pressure over many hours. These signals are easily distorted by volitional inputs, movement and other types of physical interference. Consequently, the analysis of airway signals using time‐series techniques is often hampered by random and difficult to detect signal aberrations. An alternative to time‐dependent methods is the application of Fourier analysis to assess variations in periodicity from its frequency spectrum. The approach of spectral analysis in sleep has been applied to the EEG for sleep staging (Campbell 2009) and to ECG signals in describing HRV (Chouchou and Desseilles 2014).

**Figure 1:**
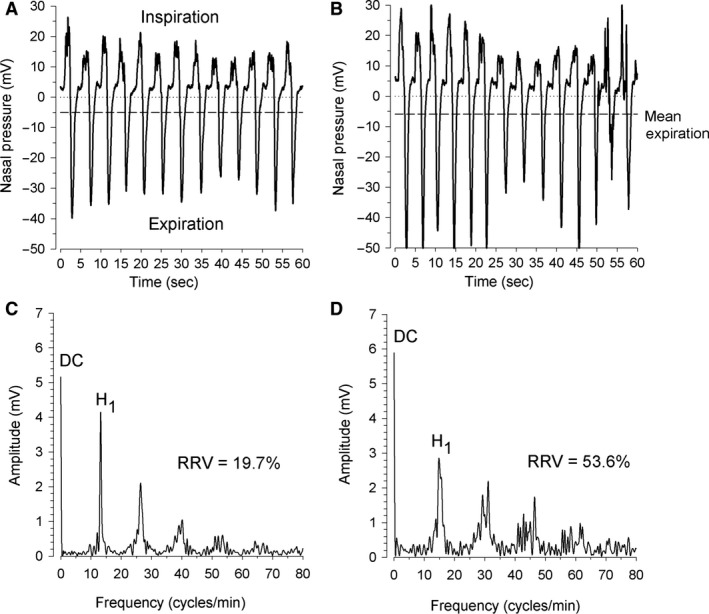
Nasal pressure signals and corresponding frequency spectra measured in a subject during sleep stage N2 (graph A) and REM sleep (graph B). The mean expiratory values (dashed lines) equal the spectra's DC components. The first harmonic peak (H_1_) is located at the fundamental frequency or mean respiratory rate of the subject during the time‐window. With increased variation in breath‐to‐breath timing, the power near the fundamental frequency spreads across neighboring frequencies, leading to a widening and reduction in peak height.

Spectral analysis of airway flow has been used to characterize patient‐ventilator asynchrony (PVA) in critically ill, mechanically ventilated patients (Gutierrez et al. 2011). The method consists of sampling airway flow during a defined time period, followed by the application of Fourier analysis to generate its frequency spectrum. During regular breathing, the frequency spectrum of airway flow is characterized by sharply defined peaks of progressively lower amplitude located at multiples of the mean respiratory rate frequency (Fig. 1). The amplitude ratio of the first harmonic peak (H_1_) to that of zero, or DC frequency peak (H_1_/DC) has been used as a measure of spectral organization. H_1_/DC varies inversely with PVA, with values <40% being associated with asynchronous breathing (Gutierrez et al. 2013).

The two main types of sleep are rapid‐eye‐movement (REM) and nonrapid‐eye‐movement (NREM). The latter includes three distinct stages: N1, N2, and N3. As sleep progresses from stage N1 to N3, brain waves become slower and more synchronized. Stage N3 is referred to as “deep” or “slow‐wave” sleep. In this study, we explored the feasibility of applying spectral analysis to airway flow data gathered during all‐night polysomnography (PSG) in spontaneously breathing individuals. We used the nasal pressure transducer signal as a proxy for airway flow (Grover and Pittman 2008), and generated sequential frequency spectra at defined time intervals for the duration of the PSG. Our aim was to detect and quantitate RRV during the various sleep stages, including episodes of wakefulness, in individuals with no obstructive sleep apnea (OSA).

## Materials and Methods

We analyzed all PSG records (Respironics Alice 6 LDXN Sleep Diagnostic System, Philips Respironics, Murrysville, PA) of patients tested at the Center for Sleep Disorders at the Medical Faculty Associates at George Washington University from 1 September 2014 through 31 August 2015. The study was approved by The George Washington University Institutional Review Board (No. 091546) who waived informed consent provided all data were de‐identified.

### Experimental set‐up

An adult nasal pressure transducer cannula (Salter Labs, Arvin, CA) was placed according to American Academy of Sleep Medicine (AASM) standards of practice. Another qualitative measure of airflow was provided by a single channel oral/nasal thermistor (ProTech #1078754; Philips Respironics) with two probes taped directly under the nares and another extending to the front of the mouth (Pennock 1992). Nasal pressure and thermistor tracings were sampled for the entire duration of the PSG at 100 Hz. Arterial O_2_ saturation (S_p_O_2_) was measured by pulse oximetry (MasimoSET Radical, Masimo, Irvine, CA) and sampled at 1 Hz. The subject's body position during sleep was assessed by an integrated microelectromechanical system based accelerometer (Philips Respironics) with manual‐override by the attending technologist. Data were digitally recorded on an hourly basis as European Data Format (EDF) files and exported to ASCII file format using the EDF Browser 1.56 program (Free Software Foundation, Inc., Boston, MA; General Public License).

We used the AASM definition of apnea as a ≥90% decrease in airflow signal for ≥10 sec and hypopnea as a ≥30% decrease in airflow signal for ≥10 sec with a reduction in S_p_O_2_ ≥3% or an associated arousal (Berry et al. 2015) and included in the study all patients with an apnea‐hypopnea index (AHI) <5.

### Spectrum generation and analysis

We applied the method of Gutierrez et al. (2011) to generate frequency spectra from the nasal pressure signal using software developed in‐house for the purposes of the study (Visual Basic, Microsoft Corporation, Redmond, WA). In brief, the nasal pressure signal was modified by setting all positive values to zero, resulting in a periodic signal containing only the expiratory portion of the breathing cycle. A frequency spectrum was generated by applying the Cooley–Tukey Fast Fourier Transform (FFT) algorithm (Duhamel and Vetterli 1992) to the modified nasal pressure signal data encompassed by a predetermined time‐window of constant duration. The length of the time‐window was mandated by the FFT requirement of 2^*n*^ samples per frequency spectrum. We chose 2^14^ or 16,384 samples, resulting in a time‐window of 2.73 min at sampling rate of 100 Hz. The resulting spectrum had a definition of 0.37 cycles per minute. Successive spectra were generated for each time‐window from the time data recording began (lights off) to PSG end (lights on). There were approximately 160 spectra generated for each patient during a typical all‐night PSG.

We determined the amplitude and frequency of H_1_ with a peak detection algorithm and calculated H_1_/DC for each spectrum. Spectra having H_1_/DC <15% were rejected from analysis, as preliminary data analysis showed these low H_1_/DC values to occur whenever the nasal pressure transducer signal either malfunctioned or the transducer fell off the patient's nose. The EDF files corresponding to all rejected spectra were visually inspected to confirm the absence of the pressure signal. Subjects in whom technical malfunction resulted in absent nasal pressure tracings for >50% of PSG time were excluded from the study. The spectrum's H_1_ frequency, or fundamental frequency, was taken as the patient's mean respiratory rate (RR)_tw_ during the spectral time‐window. We also computed mean S_p_O_2_ for each time‐window. For the purposes of this study, we defined respiratory rate variability as RRV = 100 − H_1_/DC%. Each spectrum was associated with its unique RRV, (RR)_tw_ and mean S_p_O_2,_.

### Synchronization of the spectra to sleep stage

A standardized scored event‐marker report was developed for each PSG containing multiple time‐stamped events (Berry et al. 2015) corresponding to the occurrence of a predefined events (i.e., apnea, hypopnea, desaturation, arousal, etc.). Each event was associated with one of four sleep stages (REM, N1, N2 and N3) or wakefulness (Wake) as identified by the AASM Manual for the Scoring of Sleep and Associated Events (Anonymous 1999). Given the random timing of these event marks, substantial time‐gaps often appeared from one mark to the next during which the sleep stages were not reported. To identify a sleep stage corresponding to each time‐window, we placed additional event marks at 5 min intervals from beginning to end of the PSG that were associated with a unique sleep stage. In this manner, all spectra were assured to span at least one unique sleep stage. The modified score event marker for each PSG was saved as an Excel worksheet (Microsoft Corporation).

We synchronized the spectral time‐windows to the scored event marker by matching each subject's EDF file initial recording time to the event marker lights off time. In this manner, each spectrum could be assigned a sleep stage. In those cases where the spectral time‐window encompassed two or more sleep stages, the spectrum was associated with the stage of longest duration. We tested the internal validity of this process for each subject by comparing the total time spent in various sleep stages, as reported in an epoch‐by‐epoch analysis of the PSG (PSG time), to the product of the spectral time‐window (2.73 min) and the number of spectra associated with each sleep stage (Spectral time).

For each patient's PSG, we calculated the mean values for RRV, (RR)_tw_, S_p_O_2_ and the time spent in each sleep stage and wakefulness.

### Statistics

Data distributions were examined and non‐normal distributions were either natural‐log transformed if positively skewed, or recoded into quartiles if outliers were present. Descriptive statistics (mean, standard deviation, median, intraquartile range) are reported by sleep stage. As all data are examined across sleep stages within subjects, and because not all subjects entered every sleep stage, leading to missing data, data analysis required an approach that both accounts for auto‐correlation within subjects, and that does not drop subjects from analysis if they are missing data at a single sleep stage. Both analysis of variance (ANOVA) and repeated‐measures ANOVA are thus ruled out. Therefore, mixed model regression was used to examine univariate associations between physiological variables and sleep stage. As we were interested in within‐subject changes in RRV parameters across sleep stages, a fixed‐effects mixed model was used, which compares values across stages within subjects. First, univariate models were tested for each individual physiological variable (total sleep time per stage, breathing rate, H_1_/DC, S_p_O_2_) using Wake as the reference group, and then using REM as the reference group. Next, using H_1_/DC as the time‐varying dependent variable, models were tested adjusting for total time and breathing rate at each sleep stage, to examine the independent effects of sleep stage on RRV, adjusting for time‐varying covariates. SAS version 9.3 (Cary, NC) was used for all data analysis, with *P* < 0.05 considered significant.

## Results

Of 492 diagnostic PSG records performed in 1 year, we identified 42 that met the inclusion criterion of AHI <5. We excluded from analysis four patients whose nasal pressure tracings were measured for <50% of total PSG time due to technical factors. The cohort's median age was 40.0 [32.3–55.5] years with BMI of 24.5 [22.1–31.7] kg m^−2^. There was a preponderance of women (76.3%). The ethnic composition, prevalence of comorbid conditions and use of medicines, alcohol and tobacco are typical of the adult patient population served by our institution (Table 1).

**Table 1:** Ethnicity, comorbidities and current use of medications, alcohol and tobacco (*n* = 38)

Ethnicity (%)	
Black	47.4
White	36.8
Hispanic	5.3
Asian	7.9
Other	2.6
Major comorbidities (%)	
Anxiety/depression	26.3
Lung disease	23.7
Hypertension	21.1
CVA history	10.5
Diabetes mellitus	10.5
Cardiac disease	5.3
Medications/substance use (%)	
Alcohol	18.4
Tobacco	13.2
SSRI	21.1
Pain medications	23.7

CVA, cerebrovascular accident; SSRI, selective serotonin reuptake inhibitor.

Figure 1 shows nasal pressure signals with their corresponding frequency spectra measured in a subject during sleep stage N2 (graph A) and REM sleep (graph B). The nasal pressure mean expiratory values (dashed lines) equal the spectra's DC components. The first peak (H_1_) is located at the fundamental frequency or mean respiratory rate of the subject during the time‐window.

**Figure 2:**
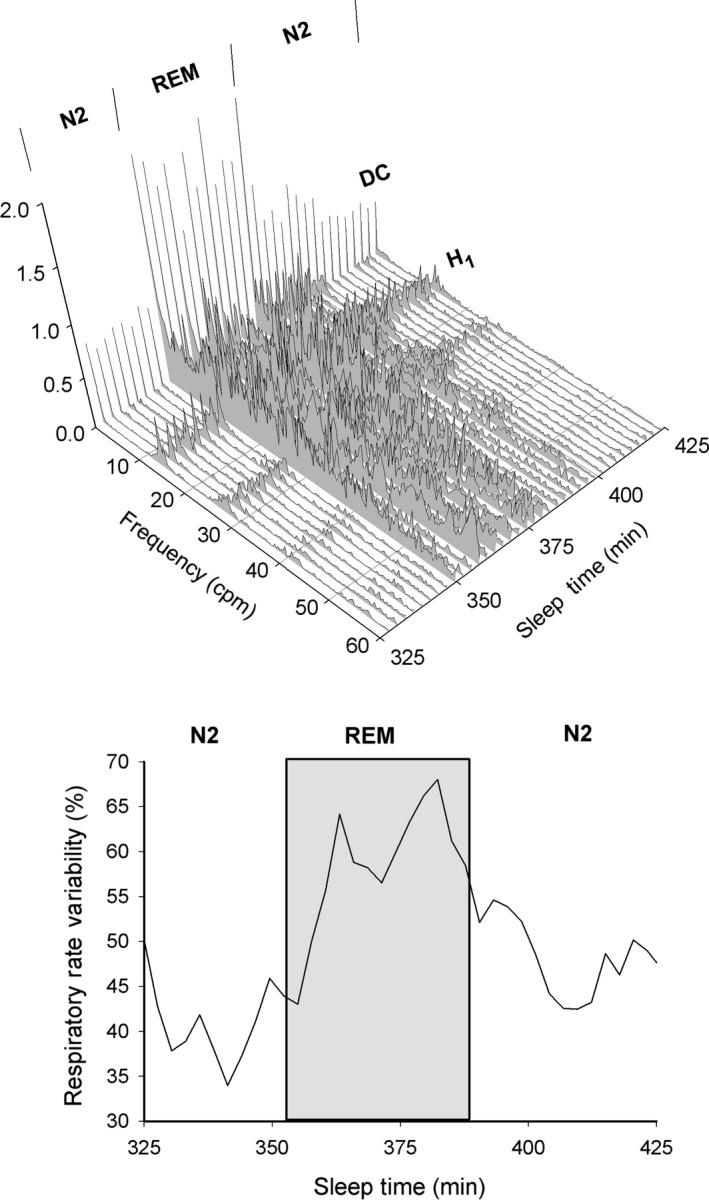
(Top) Consecutive waterfall plots of expiratory nasal pressure spectra obtained from a single patient during N2 and REM sleep stages. (Bottom) Corresponding respiratory rate variability (RRV = 100 − H_1_/DC%) measured from the spectra. See text for details.

Greater breath‐to‐breath timing variation causes the power near the fundamental frequency to spread out across neighboring frequencies, leading to a reduction in peak height as the peaks broaden and the high‐frequency harmonics disappear. Conversely, mean expiratory flow (and consequently DC amplitude) changes little during the time‐window. The result is an increase in RRV, expressed in term of 100 − H_1_/DC%.

Using spectral analysis, alterations in nasal pressure frequency spectra can be followed sequentially. This is illustrated in Figure 2 that shows a waterfall plot of spectra determined at consecutive 2.73 min time‐windows for 100 min of sleep time. Initially, the spectra display a regular peak pattern typical of steady breathing with the subject in stage N2. As the subject enters REM, there is a significant increase in amplitude, reflecting greater nasal airflow, with noticeable spectral disorganization. The spectra revert to their initial pattern with return to N2. A numerical assessment of spectral changes is shown in the bottom graph, where RRV (computed as 100 − H_1_/DC %) is plotted against time. RRV increases as the subject moves from N2 to REM (shaded box). Mean RRV values during these sleep stages is 41.2% for N2 and 60.0% for REM. As the subject reverts to N2, the mean RRV decreases to 48%.

For the cohort, the PSG duration was 434 ± 71 min, with a sleep efficiency of 80.5 ± 13.1%. A total of 6084 spectra were generated for the group, or 160 ± 26 spectra per subject. We rejected 36 spectra due to an absence of nasal pressure signal, or 0.6% for the group. Table 2 shows the percent of total sleep time for the various sleep stages obtained from the PSG (PSG time) and that calculated by multiplying 2.73 min by the number of spectra in a given sleep stage (Spectral time). The mean absolute difference was <2% for all stages confirming the validity of the method used to assign a given sleep stage to a spectrum. The percent time distribution according to sleep stage was typical of individuals without OSA (Aeschbach et al. 1996), with the majority of sleep time occurring in N2.

**Table 2:** Time spent during various sleep stages as percent of total sleep time obtained directly from the PSG (PSG time) and compared to that calculated using the spectra (spectral time). See text for explanation. (*n* = 38) There were no significant differences between times spent in any of the sleep stages

Sleep stage	PSG time (%)	Spectral time (%)	Absolute difference (%)
REM	17.4 ± 7.6	17.1 ± 7.8	0.3 ± 2.5
N1	3.6 ± 4.2	4.1 ± 3.3	0.5 ± 3.9
N2	63.7 ± 14.6	62.5 ± 10.0	1.2 ± 3.7
N3	15.2 ± 14.6	17.3 ± 15.9	1.6 ± 5.3

PSG, polysomnography; REM, rapid‐eye‐movement.

Compared to Wake, RR was lower for all sleep stages (*P* < 0.01 for N1 and N3; *P* < .0001 for REM and N2) (Table 3). There were no differences in RR among the sleep stages. Also when compared to Wake, there was a statistically (*P* < 0.01), although not clinically significant decline in S_p_O_2_ during all sleep stages (Table 3).

**Table 3:** Sleep time, respiratory rate (RR) and arterial O_2_ saturation (S_p_O_2_) measured by pulse oximetry

Parameter	Wake (*n* = 38)	REM (*n* = 36)	N1 (*n* = 35)	N2 (*n* = 38)	N3 (*n* = 34)
Sleep time (min)	85.5 ± 63.3	65.9 ± 31.5	15.3 ± 11.4* ^,^ §	215.6 ± 66.3* ^,^ §	64.8 ± 52.8
RR (bpm)	16.8 ± 2.4	15.2 ± 3.0*	15.7 ± 2.8†	15.5 ± 2.2*	15.9 ± 2.4†
S_p_O_2_ (%)	97.1 ± 1.2	97.0 ± 1.2‡	96.7 ± 1.5‡	96.6 ± 1.5†	96.2 ± 1.2* ^,^ ¶

REM, rapid‐eye‐movement. *P < .0001 versus Wake. †*P* < .01 versus Wake. ‡*P* < .05 versus Wake. §*P* < .0001 versus REM. ¶*P* < .01 versus REM.

**Figure 3:**
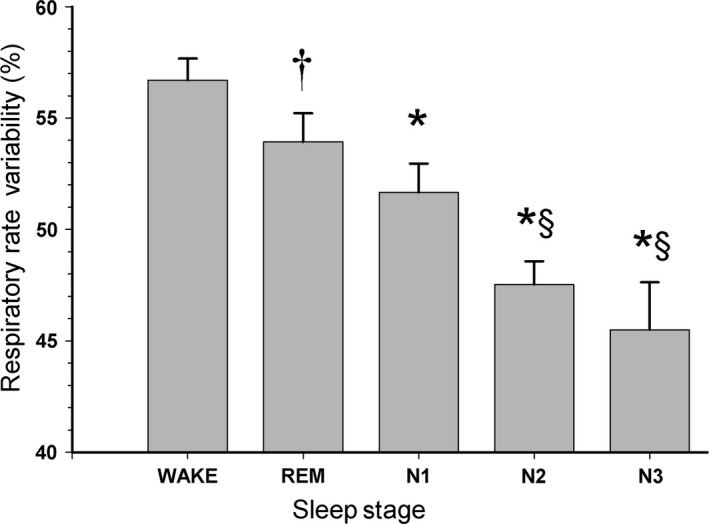
Respiratory rate variability (RRV) for patients (*n* = 38) with apnea‐hypopnea index <5 shown according to sleep stage and wakefulness. RRV was calculated from the expiratory components of the nasal pressure signal and expressed as 100 − H_1_/DC%. RRV was greatest during Wake and rapid‐eye‐movement (REM) and decreased progressively with depth of sleep from N1 to N3. *P < 0.0001; †*P* < 0.05 compared to Wake; §*P* < 0.001 compared to REM; mean ± SEM.

Figure 3 shows RRV for Wake and the various sleep stages. RRV was greatest during Wake and REM and decreased progressively with depth of sleep from N1 to N3 (*P* < 0.0001). RRV was lower during REM, compared to Wake (*P* < 0.05). Adjusting for time and respiratory rate in each stage, RRV remained significantly lower in all sleep stages, compared with Wake (*P* < 0.0001 for N1, N2, and N3, *P* = 0.0033 for REM), and was significantly lower during N2 (*P* = 0.0024) and N3 (*P* < 0.0001), compared to REM. The subjects spent the majority of their time in the supine position (Table 4), but there were no differences in RRV according to body position.

**Table 4:** Percent time spent in different body positions during sleep and associated average respiratory rate variability (RRV) value (*n* = 38)

	Time (%)	RRV (%)
Supine	48.7 ± 32.4	53.0 ± 6.6
Prone	6.4 ± 18.0*	50.2 ± 11.4
Right	28.7 ± 22.7†,‡	51.4 ± 7.5
Left	16.2 ± 23.5*	51.2 ± 8.6

*P < 0.01 compared to Supine; †*P* < 0.05 compared to Supine; ‡*P* < 0.01 compared to Prone; mean ± SD.

## Discussion

The physiological response of the respiratory system during wakefulness differs from that of sleep (Dempsey et al. 1996). As volitional control and wakeful stimuli wane, ventilation becomes a function of the metabolic rate during sleep (Sowho et al. 2014). Chemoreceptor sensitivity is diminished, this being more pronounced during REM sleep, resulting in an attenuated responsiveness to chemical and mechanical stimuli (Douglas et al. 1982b; Hedemark and Kronenberg 1982; Dempsey and Smith 2014). Although there is marked variability among normal individuals in upper airway collapsibility (Wiegand et al. 1989), reduced tonic drive of pharyngeal dilator muscles during sleep increases upper airway resistance during inspiration (Henke et al. 1990). These alterations are bound to influence the pattern of respiration during sleep.

Here, we present data from 38 patients with no OSA in whom we measured RRV during sleep. We quantitated RRV during the sleep stages by applying a previously validated method of spectral analysis to the expiratory portion of the nasal pressure signal. We found differences in RRV between wakefulness and REM sleep, and also noted that RRV decreased in all NREM stages in concert with the depth of sleep, being lowest in N3. These findings are consistent with the long held observation that respiration becomes more regular during sleep (Reed and Kleitman 1926).

We analyzed only the expiratory portion of the nasal pressure signal. This choice was dictated by the absence of a DC component in the spectrum of the entire signal, since airway flow during the respiratory cycle approximates zero. Furthermore, H_1_ depends not only on RRV, but also on signal strength, thus preventing the accurate comparison among spectra based solely on H_1_ morphology. Conversely, the expiratory component of the nasal pressure signal has a nonzero mean value and its associated spectrum has a measurable DC component that also changes with signal strength. The amplitude of the DC component thus provides a metric with which to assess changes in H_1_, that is, independent of signal strength. Although other pattern recognition schemes could be used to assess spectral changes, the amplitude ratio H_1_/DC is known to be a reliable parameter of spectral organization (Gutierrez et al. 2011), one that allows for the comparison of RRV among sleep stages and across patient groups.

There is a paucity of studies in the literature reporting RRV in sleep. Shore et al. (1985) compared the coefficient of variation (CV) of the respiratory rate of healthy young and elderly individuals from respiratory inductive plethysmographic (RIP) signals. They obtained 15 min spans of data during wakefulness and sleep and determined breathing periodicity by visual examination of the data. CV was similar for wakefulness and a combined stage N1–2, but declined during N3. These investigators (Pack et al. 1988) subsequently sampled RIP signals at a relatively low rate of 2.5 Hz and applied digital comb filtering to determine ventilatory periodicities in eight individuals without OSA aged over 66 years. Unlike their prior study, they found greater periodicities in N1–2 compared to wakefulness. In agreement with our results, they found periodicities in N3 to be mostly absent. Both studies, however, lacked data measured during REM. Immanuel et al. (2014) also used RIP to study RRV in children with sleep disordered breathing and found both groups to have greater RRV during REM when compared to stages N2 and N4.

Rostig et al. (2005) and Kantelhardt et al. (2003) used the technique of detrended fluctuation analysis to measure breath‐to‐breath variability in healthy individuals from airway flow measured with a pneumotachograph. Also in agreement with our findings, they noted greater RRV in REM when compared to NREM sleep. More recently, Nguyen et al. (2016) proposed the use of a semi‐automated technique to characterize RRV from the statistical properties of the time between successive breaths, or interbreath interval (IBI). They identified the zero‐crossing and peak inspiratory and expiratory points of airway flow with an amplitude threshold algorithm and computed successive IBIs. RRV was characterized by the SD and CV of the IBIs. Their results are similar to ours, in that RRV was noted to be greater during REM and wakefulness compared to NREM.

We noted a decrease in respiratory rate during all sleep stages compared to wakefulness, but no differences in respiratory rate among the sleep stages. These results contrast with those of Douglas et al. (1982a), who found increases in RR during sleep in 19 healthy men in whom airway flow was measured via a fiberglass mask connected to a hot wire anemometer. Other studies in young, healthy men, however, have found either no change (Stradling et al. 1985) or decrease (Tabachnik et al. 1981; Krieger et al. 1983; Lopes et al. 1983) in RR with sleep compared to wakefulness. Stradling et al. (1985) argued that the increase in RR during sleep, as reported by Douglas et al., may have been related to an imposed additional dead space and to the relative hypoxemia experienced by the subjects being studied at an elevation of 1600 m.

There are no studies on the effect of body position on RRV. On the basis of prior studies on OSA patients (Joosten et al. 2014), we expected to find greater RRV in the supine and lateral positions (Oksenberg et al. 2000). Our cohort of individuals with no OSA spent the majority of their sleep time on the supine position, but no relationship between body position and RRV was evident. It maybe that non‐OSA patients experience little or no airway diameter reduction during the supine position and therefore no increase in RRV. Further studies are needed to determine the relationship with of RRV to body position in OSA patients.

Our study has several limitations. It is a retrospective, single‐center study having a relatively small sample size. We strived to minimize retrospective bias by searching for individuals with AHI <5 in all diagnostic PSGs performed during a predefined time period of 1 year. The number patients meeting entry criteria was determined by the few PSG studies that were ordered in individuals with sleep‐related complaints, but who turned out to have no sleep apnea. Given these constraint, however, our cohort is significantly larger than other studies on RRV during sleep (Kantelhardt et al. 2003; Rostig et al. 2005; Nguyen et al. 2016).

We do not consider the subjects studied here to represent a “normal” cohort since these individuals were initially referred for a PSG in response to a sleep‐related complaint. None of the subjects, however, was considered to have OSA, as defined by an AHI <5 on PSG. This in contrast to studies that report RRV in putatively normal individuals, but include in the cohort patients with sleep disordered breathing. Krieger et al. (1983) and Shore et al. (1985) found 17.5% and 38%, respectively, of young healthy subjects without sleep‐related complaints to have AHI >5. In the study of Nguyen et al. (2016), the mean AHI of the control group was 9.9 ± 9.7. On the other hand, our heterogeneous cohort included individuals of both genders and diverse ethnic backgrounds, encompassing a wide range of age and body weight and a significant portion was afflicted with comorbidities typical of patients seen commonly in sleep disorders clinics.

**Figure 4:**
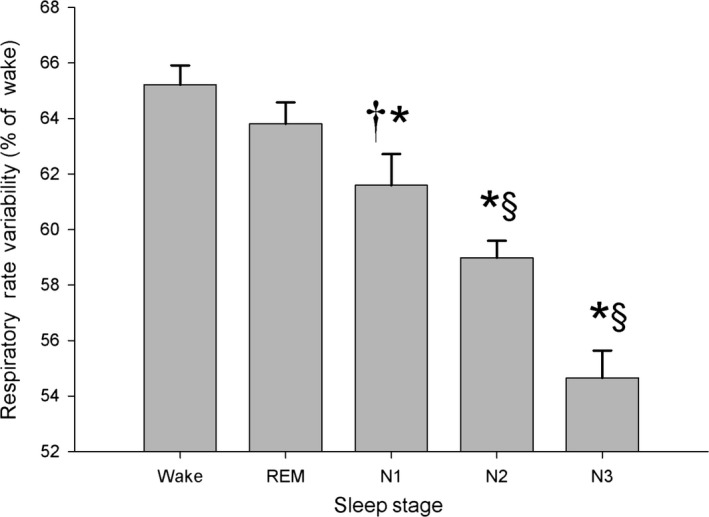
Respiratory rate variability (RRV) for patients (*n* = 38) with apnea‐hypopnea index <5 shown according to sleep stage and wakefulness. RRV was calculated from the expiratory components of the thermistor signal and expressed as 100 − H_1_/DC%. The results are similar to those noted when using the nasal pressure signal, RRV was greatest during Wake and REM and decreased progressively with depth of sleep from N1 to N3. *P < 0.0001; †*P* < 0.05 compared to Wake; §*P* < 0.001 compared to REM; mean ± SEM.

We used the nasal pressure transducer signal as a surrogate for airway flow (Grover and Pittman 2008). This is a clinically accepted technique (Thurnheer et al. 2001) and the relationship between nasal pressure and flow has been previously described (Wheatley et al. 1991). Nasal pressure transducers, however, record only nasal airflow and it is possible for the signal to lose power with oral breathing. Since spectral analysis can be applied to any periodic signal associated with respiratory motion, we compared RRV calculated from the expiratory components of both nasal pressure and thermistor signals (BaHammam 2004). Figure 4 shows RRV calculated from the thermistor signal. There were no differences in the relative degree of RRV among sleep stages when calculated using nasal pressure or thermistor signal. We prefer, however, to use the expiratory portion of the nasal pressure signal, since it appears to be more reliable.

One strength of our study is the use of minimally invasive measures on ventilation, therefore avoiding possible alterations in respiratory patterns produced by masks or other devices designed to measure flow. Furthermore, the lack of selective pre‐analysis of the data by smoothing or digital filtering adds to the robustness of the method. The application of spectral analysis, instead of a time‐dependent technique of time‐series analysis, bypassed the need to apply digital filtering minimizing raw data manipulation. Instead of evaluating selected, noise‐free time periods, we analyzed data as they were recorded, producing a spectrum every 2.73 min for the duration of the PSG. The ability to analyze airway signals directly may potentially allow for the determination of RRV concurrently with the acquisition of PSG data on a real‐time basis.

The physiological consequence of RRV in sleep, in particular during pathological states, is an open field for investigation. For instance, recent studies have shown that breathing patterns differ in mixed central and obstructive apnea compared to obstructive dominant apnea (Yamauchi et al. 2013) with a possible association between CPAP acceptance and breathing irregularity. Research in RRV during sleep has been hampered previously by the lack of a robust and reliable measuring technique. The application of spectral‐frequency analysis to the pressure nasal airflow signal appears to be a novel and promising method with which to assess RRV during sleep. It remains to be tested if the method is capable of differentiating normal from pathological sleep patterns.

## Conflict of Interest

None declared.
